# „Wohlfahrtsstaat und Infrastruktur – das Soziale organisieren“

**DOI:** 10.1007/s12592-022-00420-w

**Published:** 2022-07-26

**Authors:** Sigrid Leitner

**Affiliations:** Technische Hochschule Köln, Gustav-Heinemann-Ufer 54, 50968 Köln, Deutschland

**Keywords:** Sozialversicherungen, Grundsicherung, Soziale Arbeit, Daseinsvorsorge, Soziale Infrastruktur, Social insurance, Welfare benefits, Social work, Maintenance, Social infrastructure

## Abstract

Der deutsche Sozialstaat organisiert soziale Sicherung traditionell auf zwei höchst unterschiedliche Weisen: Zum einen über die erwerbsarbeitszentrierten staatlichen Sozialversicherungen, die typische Risiken im Lebenslauf für alle Erwerbstätigen und ihre Familien absichern, zum anderen über eine bedarfsgeprüfte Existenzsicherung und individualisierte Hilfen im Einzelfall. Diese wohlfahrtsstaatliche Komplementärkonstruktion ist durch sozioökonomische Veränderungsprozesse unter Druck geraten: Ein verringertes Wirtschaftswachstum, steigende Arbeitslosigkeit und die Alterung der Gesellschaft führten zu steigenden Ausgaben in der Sozialpolitik, die nicht durch ein entsprechendes Einnahmenwachstum kompensiert werden konnten. Sowohl das Sozialversicherungs- als auch das Grundsicherungssystem wurde entsprechend angepasst, wobei es nicht nur um die Kürzung von Sozialleistungen ging, sondern auch die normativen Sicherungsziele neu ausgerichtet wurden. Der reformierte Sozialstaat setzt auf Aktivierung, Eigenverantwortung und Selbstbestimmung – und zwar in allen Bereichen. Damit stellt sich auch grundsätzlich die Frage, inwiefern der deutsche Wohlfahrtsstaat in seiner Gesamtheit noch in der Lage ist, soziale Problemlagen aufzufangen. Aus einer ganzheitlichen Perspektive wird dafür plädiert, die Fragmentierung des sozialen Sicherungssystems zu überwinden und auf Basis von konkreten Problemkonstellationen die Organisation des Sozialen neu zu denken.

## Abstract (en)

The German welfare state organizes social policy traditionally in two distinct ways. On the one hand, work-related and state-organized social security schemes protect the working population and their families from typical social risks during the life course. On the other hand, means-tested welfare benefits and individualized social services support individuals outside the labor market. Due to socioeconomic change, this complementary construction of the welfare state is under increasing pressure: declining economic growth rates, increasing unemployment rates, and the aging population lead to expanding costs for the welfare state, whereas funding for social policy shrinks. Social security benefits as well as welfare benefits were adapted accordingly in terms of benefit reductions but also by readjusting the normative aims of the German welfare state. The new paradigms of activation, self-responsibility, and autonomy can be found in all social programs. Thus, the basic question is whether the reformed welfare state as a whole is still able to deal with the social problems at hand. From a holistic point of view, it is argued that the fragmentation of the social protection system should be overcome in order to develop an integrated perspective of how to organize social policy.

Das Ausmaß an gesellschaftlicher Solidarität lässt sich unter anderem an den Strukturen staatlicher Sozialpolitik ablesen. Ein generöses Sozialleistungssystem mit hohem Umverteilungseffekt in Kombination mit einem ausgebauten Sozialdienstleistungssektor führt zu geringerer soziale Ungleichheit als ein streng bedarfsgeprüftes, in seiner Leistungshöhe kaum existenzsicherndes und marktbasiertes Armutsregime, welches die soziale Polarisierung der Gesellschaft reproduziert. In der vergleichenden Wohlfahrtsstaatsforschung wird ersteres Szenario üblicherweise mit dem sozialdemokratischen und letzteres mit dem liberalen Idealtypus eines „welfare regime“ (Esping-Andersen 1990) assoziiert. Die Frage, wie das Soziale organisiert wird, entscheidet somit über die Art und das Ausmaß sozialer Ungleichheit einer Gesellschaft und ist Ausdruck (tradierter und gleichzeitig wandelbarer) wohlfahrtskultureller Grundüberzeugungen.

Lange Zeit wurde in der (deutschen) Sozialpolitikforschung der Fokus auf die Sicherung von typischen sozialen Risiken im Lebenslauf (Kaufmann 1973) gerichtet: Krankheit, Unfall, Alter, Arbeitslosigkeit und später auch Pflegebedürftigkeit. Für diese Risiken stehen generalisierte Leistungen zur Verfügung: Die fünf Sozialversicherungszweige bilden das Herzstück des deutschen Wohlfahrtsstaats wie auch der Wohlfahrtsstaatsforschung (vgl. Kaufmann et al. 2016). Dabei ist innerhalb dieses *ersten* sozialen Netzes wiederum eine Priorisierung der Geld- bzw. Lohnersatzleistungen festzustellen, während von der Sozialversicherung finanzierte (gesundheitsbezogene) Sach- und Dienstleistungen zur Regeneration der Arbeitskraft nur zweitrangig Berücksichtigung finden. In dieser Makroperspektive auf den Wohlfahrtsstaat stehen vor allem Fragen nach den gesamtgesellschaftlichen Verteilungs- und Steuerungswirkungen im Vordergrund, es geht im Kern um soziale Ungleichheits- und Wirkungsforschung. Das Sozialversicherungssystem beruht auf der Solidargemeinschaft der Versicherten und erzeugt über seine Regelungsmechanismen entsprechende Ein- und Ausschlüsse.

Das *zweite* soziale Netz, welches die bedarfsgeprüfte Existenzsicherung über Grundsicherungsleistungen sowie die Hilfe in individuellen Problemlagen über die Erbringung sozialer Dienstleistungen umfasst, wurde zwar als notwendige Ergänzung der Sozialversicherungspolitik betrachtet, war jedoch vom Umfang des finanziellen Leistungsaufwands her aus einer Makroperspektive vergleichsweise weniger bedeutsam. Die Bearbeitung von Einzelfällen ist traditionell zudem auf der Ebene der kommunalen Sozialpolitik angesiedelt, welche ein heterogenes Feld mit unübersichtlicher Datenlage darstellt und deshalb rein forschungspragmatisch oftmals nicht attraktiv erscheint. Erst mit zunehmenden Kostensteigerungen und damit einhergehenden Fragen der Bedarfs- und Verteilungsgerechtigkeit im Grundsicherungsbereich (Stichwort: Hartz IV) sowie mit dem Erstarken der Sozialen Arbeit als Profession und wissenschaftliche Disziplin kam es zu einer Belebung der einzelfallbezogenen Mikro- und organisationsbezogenen Mesoperspektive der Sozialpolitikforschung. Sowohl der Adressat*innenbezug als auch die Inblicknahme des organisationalen Settings der Dienstleistungserbringung brachten neue Forschungsfragen mit sich.

Eine Makro‑, Meso- und Mikroebene integrierende Perspektive auf den „Sozialstaat als kritische Infrastruktur“ wird erst in jüngster Zeit und nicht zuletzt im Zuge der COVID-19-Pandemie entwickelt (DIFIS 2022). Die Bedeutung sozialpolitischer Strukturen für die Selbsterhaltung der Gesellschaft wurde mit dem Begriff der „Systemrelevanz“ markiert. Stellt man den Blick weit, geht es sowohl um Strukturen der Daseinsvorsorge im engeren (Wasser- und Energieversorgung, Abfallwirtschaft, öffentlicher Personennahverkehr) und weiteren Sinn (stationäre Versorgung durch Kinderbetreuungseinrichtungen, Schulen, Krankenhäuser, Pflegeeinrichtungen etc. sowie ambulante soziale Dienstleistungen) als auch um alle Arten von sozialpolitischen Sach- und Geldleistungen. Für eine integrierte Perspektive auf die Organisation des Sozialen kann es hilfreich sein, von konkreten Problemkonstellationen aus und multiperspektivisch zu denken, um Systemgrenzen zu überwinden und ganzheitliche Lösungen zu finden.

Der folgende Beitrag greift zunächst als historische Grundlegung der Organisation des Sozialen das nationalstaatlich geprägte Sozialversicherungsprinzip (Abschn. 2) und die ergänzende Organisationsebene der kommunalen Sozialpolitik (Abschn. 3) auf.^1^ Danach werden Problematiken der Organisation des Sozialen diskutiert: Das Ende des „golden age of the welfare state“, das Neue Steuerungsmodell der Kommunen, die Hinwendung zum „adult worker model“, die Diversifizierung des männlichen Ernährermodells sowie die Neujustierung und Neuerfindung des Sozialen (Abschn. 4). Abschließend werden Konturen einer integrierten Perspektive auf das Soziale im Sinne eines zukünftigen Forschungsprogramms formuliert.

## Historische Grundlegung I: Sozialversicherung als nationales Organisationsprinzip

Die historischen Wurzeln der deutschen Sozialversicherung liegen in ersten vereinzelten Versicherungen in gewerkschaftlicher, betrieblicher und berufsständischer Trägerschaft. Erst mit der Einführung der staatlichen Sozialversicherung unter Reichskanzler Otto von Bismarck und Kaiser Wilhelm II wurde die Absicherung von Erwerbsrisiken Ende des 19. Jahrhunderts zu einem staatlichen Aufgabenbereich. Dabei wurden zunächst die Risiken des Einkommensausfalls bei Krankheit, Unfall, Alter und Invalidität abgesichert. Im Fokus standen die als Folge von Industrialisierung und Urbanisierung von Pauperismus geprägten Lebensbedingungen der Industriearbeiterschaft und die Bedrohung der Stabilität des politischen Systems durch die soziale Frage, die sich in einer sich zunehmend organisierenden Arbeiterbewegung widerspiegelte. Mit der sprichwörtlich gewordenen zweigleisigen politischen Strategie von „Zuckerbrot und Peitsche“ (Sozialversicherung und Sozialistengesetz) sollte die Industriearbeiterschaft durch eine Verbesserung ihrer Lebensbedingungen befriedet, die oppositionelle sozialdemokratische und katholische Arbeiterbewegung unterdrückt und die innere Reichsgründung vorangetrieben werden. Gleichzeitig wurden mit der Einführung einer staatlich organisierten Pflichtversicherung die gerade im Entstehen begriffenen zivilgesellschaftlichen Solidarkassensysteme zurückgedrängt. Der Staat positionierte sich damit als wichtiger – und mächtiger – Garant sozialer Sicherheit im deutschen Wohlfahrtsregime (vgl. grundlegend dazu: Tennstedt 1981; Reidegeld 1996). Betriebliche und private Versicherungen spielten in der Folge nur eine marginale Rolle.

Organisationstheoretisch interessant ist die von Anfang an tripartistisch angelegte Konstruktion der Sozialversicherungen: Arbeitnehmer*innen und Arbeitgeber*innen beteiligen sich – mit Ausnahme der Unfallversicherung – paritätisch an der Finanzierung durch eine lohnbezogene Beitragsleistung, und der (paternalistisch agierende) Staat übernimmt die Finanzierungslücke bzw. Ausfallhaftung. Dies ist ein eindeutiger Hinweis darauf, dass alle Beteiligten ganz klare Vorteile aus dem Sozialversicherungssystem generieren: Geld- und Sachleistungen für die Arbeitnehmer*innen, der Erhalt der Arbeitskraft für die Arbeitgeber*innen und die Stabilisierung bestehender Machtverhältnisse für den Staat. Die anfängliche Begrenzung des Versichertenkreises auf die Industriearbeiterschaft mit geringem bis mittlerem Einkommen zeigt zum einen, dass es tatsächlich zunächst im Kern um die Verbesserung der sozialen Lage der Industriearbeiter*innen ging, da von ihnen die größte *Bedrohung* für den Staat auszugehen schien. Zum anderen ist auch interessant, dass offensichtlich für diejenigen Industriearbeiter*innen mit höherem Einkommen keine staatliche Sozialversicherung zur Abdeckung von Erwerbsrisiken vorgesehen war. Sie wurden auf ihre eigenständige Vorsorgeverpflichtung verwiesen. Diese Differenzierung nach Einkommen wirkt im Übrigen bis heute nach in Form der dualen Organisation der (gesetzlichen und privaten) Krankenversicherung sowie in der zunehmenden Auslagerung von lebensstandardsichernder Altersvorsorge in den privaten Bereich (s. Abschn. „Das Ende des ‚golden age of the welfare state‘“).

Durch die der historischen Entstehungssituation geschuldete Verknüpfung von Sozialversicherung und Industriearbeiterschaft folgt die Logik des deutschen Sozialversicherungssystems bis heute einer konsequenten Erwerbsarbeitszentrierung. Die Gruppe, die von der Sozialversicherung geschützt wird, sind in der Regel die Erwerbstätigen. Die Versicherungsleistungen waren von Anfang an dazu gedacht, Phasen des Lohnausfalls zu kompensieren. Wer aufgrund einer Krankheit oder der Folgen eines Unfalls oder wegen seines Alters oder schwerer gesundheitlicher Beeinträchtigungen temporär oder dauerhaft nicht erwerbsfähig war, erhielt von der Sozialversicherung eine Kompensationszahlung für den entgangenen Verdienst. Diese war in ihrer Höhe vom vorangegangenen individuellen Lohnniveau abhängig und im Fall der Alters- und Invaliditätsrente auch von der Dauer der eigenen Erwerbstätigkeit über den gesamten Lebenslauf. Dies führt dazu, dass im Sozialversicherungssystem die Ungleichheit der Einkommensverteilung reproduziert wird: Wer ein hohes Einkommen hat, erhält hohe Leistungen – und umgekehrt. Der Umverteilungseffekt der Sozialversicherung ist deshalb gering. Wer nie erwerbstätig war, kann auch nicht von Sozialversicherungsleistungen profitieren. Kommodifizierung, d. h. die Integration in den Arbeitsmarkt, ist damit die Voraussetzung für Dekommodifizierung: die staatlich abgesicherte Freistellung von der Erwerbsarbeit.

Seit Einführung der ersten Sozialversicherungen in Deutschland Ende des 19. Jahrhunderts kam es zu einer pfadabhängigen Weiterentwicklung derselben: Es folgte sowohl eine schrittweise Ausweitung des Versichertenkreises auf andere Berufsgruppen als auch eine Erweiterung des Systems durch die Einführung der Arbeitslosenversicherung 1927 und der Pflegeversicherung 1995. Beide Systemerweiterungen sind bemerkenswert, da sie neue soziale Risiken in das generalisierte Sozialversicherungssystem integrieren. Der Anstieg der Massenarbeitslosigkeit in der Weimarer Republik führte zu einer neuen Wahrnehmung von Arbeitslosigkeit als strukturell verursachtes soziales Risiko, das nicht länger als individuell verschuldetes Problem betrachtet werden konnte. Jede*r war potenziell von Arbeitslosigkeit bedroht, sodass die Etablierung einer Solidargemeinschaft der Versicherten eine legitime Lösung darstellte.^2^ In ähnlicher Weise führte der stetige Anstieg an Pflegebedürftigen seit den 1970er Jahren zu einer veränderten Problemwahrnehmung von Pflegebedürftigkeit als generalisiertes soziales Risiko. Aufgrund der Alterung der Bevölkerung steigt die individuelle Wahrscheinlichkeit, pflegebedürftig zu werden. Fraglich war in dem Fall, wie die Kosten der Pflege zukünftig organisiert werden können, da die kommunalen Budgets bereits ausgereizt waren. Die Einführung der Pflegeversicherung verschob die Finanzierungslast von den Kommunen auf die Arbeitnehmer*innen und die Arbeitgeber*innen, wobei die Beteiligung der Arbeitgeber*innen höchst strittig war. Als Kompensation für den Arbeitgeberbeitrag wurde schließlich in einem politischen Kompromiss ein Feiertag – der Buß- und Bettag – gestrichen.

Die Normalitätsunterstellung, die das deutsche Wohlfahrtsregime historisch charakterisiert, geht – wie oben gezeigt – von einem *Erwerbsbürger* aus, der nach Beendigung seiner Ausbildung kontinuierlich und vollumfänglich dem Arbeitsmarkt zur Verfügung steht bis zum Eintritt des Regelrentenalters. Das Pendant zu dieser Fiktion des Erwerbsbürgers im Normalarbeitsverhältnis bildet ein traditionelles Geschlechter- und Familienleitbild, das in der feministischen Sozialpolitikforschung als „männliche Ernährerehe“ (Ostner 1995; s. auch Hinrichs 1996) bezeichnet wurde. Dabei verdient der erwerbstätige Ehemann den Unterhalt der Familie, während die nicht erwerbstätige Ehefrau sich dem Haushalt, der Kindererziehung und der Angehörigenpflege widmet. Durch die über die Institution Ehe festgeschriebene Unterhaltsverpflichtung des Ehemannes gegenüber seiner Ehefrau und den gemeinsamen Kindern entsteht ein Familienarrangement, das als „ehezentrierter Familialismus“ (Lessenich 2003) bezeichnet werden kann und als konstitutiver Bestandteil des deutschen Wohlfahrtsregimes gilt (Leitner 2013). Durch die Erwerbsarbeitszentriertheit des Sozialversicherungssystems sind nicht erwerbstätige Ehefrauen eigentlich von Sozialversicherungsleistungen ausgeschlossen. Sie können nur indirekt, über die Mitgliedschaft ihres erwerbstätigen Ehemannes, abgesichert werden. So etwa in der (gesetzlichen) Kranken- und Pflegeversicherung, wo Ehefrauen beitragsfrei mitversichert werden können und im Bedarfsfall medizinische und pflegerische Leistungen erhalten. Gäbe es die beitragsfreie Mitversicherung nicht, müsste der Ehemann als Unterhaltspflichtiger für die entsprechenden Behandlungskosten seiner Ehefrau aufkommen. So aber werden die Kosten von der Solidargemeinschaft der Versicherten getragen. Ähnlich funktioniert es auch in der Rentenversicherung: Wenn der Ehemann stirbt, erhält die Witwe eine Witwenrente, die sich nach den vorangegangenen Beitragszahlungen des Ehemannes richtet. Die Leistungsansprüche des Ehemannes gehen quasi auf die Witwe über. Wäre der Verstorbene nicht verheiratet, würden seine bereits geleisteten Beitragszahlungen in die Versichertengemeinschaft zurückfließen. Diese indirekte Absicherung von Ehefrauen stellt zusammen mit der steuerrechtlichen Möglichkeit des Ehegattensplittings^3^ eine staatliche Förderung der traditionellen Ehegemeinschaft^4^ dar und institutionalisiert eine geschlechtsspezifische Arbeitsteilung, welche die Erziehungsarbeit und die Pflegearbeit an die privaten Haushalte bindet (vgl. die grundlegenden Analysen in Kickbusch und Riedmüller 1984; und Gerhard et al. 1988). Deshalb ist in Deutschland auch der soziale Dienstleistungssektor, insbesondere im Bereich der Kinderbetreuung und Pflege, traditionell vergleichsweise schwach entwickelt. Esping-Andersen (1999, S. 55) spricht von einem „service lean welfare state“.

## Historische Grundlegung II: Kommunale Sozialpolitik

Neben der Absicherung über das erwerbszentrierte Sozialversicherungssystem und die daran geknüpfte Mitversicherung von Familienangehörigen stellt die auf der kommunalen Ebene verankerte Existenzsicherung die zweite bedeutende Schiene der staatlichen Organisation des Sozialen dar. Parallel und in Ergänzung zur kirchlichen Armenfürsorge entwickelten die deutschen Kommunen ihre eigenen (stigmatisierenden und repressiven) Systeme der Armutsbekämpfung. Bis in die 1950er Jahre lag es im Ermessen der staatlichen Behörden, ob und wie sie Bedürftigen soziale Fürsorge gewährten. Die staatliche Versorgung der Bedürftigen diente dabei vorrangig dem Schutz der öffentlichen Sicherheit und Ordnung. 1954 entschied das BVerwG, dass aus Art. 1 GG nicht nur Abwehrrechte der Menschen gegen staatliche Eingriffe, sondern auch der Anspruch auf Sicherung einer menschenwürdigen Existenz in Form von finanziellen Leistungen abgleitet werden kann. In der Folge wurde mit der Einführung des Bundessozialhilfegesetzes 1961 erstmals ein bundesweit einheitlicher Standard für die Gewährung von Grundsicherungsleistungen geschaffen. Ziel war die Einlösung der im Art. 72 GG geforderten gleichwertigen Lebensverhältnisse im gesamten Bundesgebiet.

Armut wird im deutschen Wohlfahrtsstaat nach dem Prinzip der Bedarfsgerechtigkeit *bekämpft*: Wer weder durch eigene Arbeitsleistung noch durch die Unterstützung von Unterhaltspflichtigen oder aufgrund von Sozialversicherungsleistungen ein *menschenwürdiges Existenzminimum* erreicht, erhält Grundsicherungsleistungen. Diese sind immer subsidiär, also nur nachrangig zu anderen Einkommensmöglichkeiten zu gewähren, die Prüfung des tatsächlichen Bedarfs und die vorrangige Verpflichtung zum Einsatz der eigenen Arbeitskraft sind somit konstitutive Bestandteile der Grundsicherungslogik.

Neben der finanziellen Unterstützung können bedürftige Personen auch soziale Hilfen in besonderen Lebenslagen erhalten. Somit etablierte sich mit der neuen Sozialhilfe auch eine neue Sichtweise auf die Armutsbevölkerung. Während im Fürsorgesystem des Kaiserreichs und auch noch der Weimarer Republik der Aspekt der Sozialdisziplinierung im Vordergrund stand, wurden nun die individuellen Abweichungen der Armen von der gesellschaftlichen Norm pathologisiert und darüber der Bearbeitung durch professionelle Soziale Arbeit zugeführt. Diese Abwendung von moralischen Kategorien führte zu einer neuen Adressat*innenkonzeption, die geprägt war von einem Behandlungsappell jenseits von Eigenverantwortung, Schuld und Strafe und einer Normalisierungsfiktion folgte. Die Armen sollten durch professionelle Intervention auf den *richtigen* Weg gebracht und in die Gesellschaft integriert werden (Lutz 2018).

Der Fokus der Grundsicherung wie der Sozialen Arbeit richtete sich somit bis in die 1990er Jahre hinein auf diejenigen Randgruppen der Bevölkerung, die es aus unterschiedlichen Gründen nicht geschafft hatten, einen *normalen* Lebenslauf in Bezug auf Erwerbsbeteiligung (bei Männern) oder Familiengründung (bei Frauen) zu verfolgen.

## Herausforderungen der Organisation des Sozialen und ihre Bewältigung

Die dargestellten historischen Grundlegungen bilden bis heute das Grundgerüst der Organisation deutscher Sozialpolitik. Dennoch haben sozioökonomische Veränderungsprozesse über die Jahre Einfluss auf den konkreten Bauplan der Sozialversicherungen wie auch der kommunalen Existenzsicherungsleistungen genommen. In diesem Kapitel werden zentrale Tendenzen des gesellschaftlichen Wandels und ihre Folgen für die Sozialpolitik herausgearbeitet.

### Das Ende des „golden age of the welfare state“

Nach dem Zweiten Weltkrieg entwickelte sich das Wirtschaftswachstum in Deutschland bis Ende der 1960er Jahre auf hohem Niveau und schuf die Basis für die Expansion des Wohlfahrtsstaats. Erst mit dem Ende der Vollbeschäftigung und wachsender, sich verfestigender Arbeitslosigkeit beginnt in den 1980er Jahren die öffentliche Diskussion um die Finanzierbarkeit des deutschen Sozialstaats immer lauter zu werden. Im Fokus war dabei zunächst die durch den demografischen Wandel (sinkende Geburtenrate und steigende Lebenserwartung) und Langzeitarbeitslosigkeit bedingte wachsende Schere zwischen Einnahmen und Ausgaben in der gesetzlichen Rentenversicherung (GRV), die bereits in den 1990er Jahren zu kleineren Kürzungsmaßnahmen, wie z. B. die Einführung von Abschlägen bei vorzeitiger Altersrente, führte. Als Ausweg aus der Finanzierungskrise der GRV wurde mit der Rentenreform 2001 das Rentenniveau erheblich abgesenkt. Durch die Einbeziehung des sog. Riester-Faktors in die Rentenformel verringerte sich die Standardrente um die Höhe der privaten Altersvorsorgebeiträge sowie um den Anstieg der Beitragssätze zur GRV. Wer 45 Jahre lang das durchschnittliche Einkommen verdient hat, sollte dadurch zukünftig nur noch 64,5 % (statt vorher 70 %) des aktuellen Durchschnittseinkommens als Rentenleistung erhalten. Weitere Maßnahmen führten in den Folgejahren zu einer noch stärkeren Absenkung des Rentenniveaus in der GRV, so dass derzeit als politisches Ziel formuliert wird, das Standardrentenniveau nicht unter 48 % absinken zu lassen. Das ursprüngliche, 1957 mit der großen Rentenreform institutionalisierte Sicherungsziel der Lebensstandardsicherung im Alter kann heute nur noch durch eine Kombination der gesetzlichen mit einer entsprechend ausgestatteten privaten Rente gewährleistet werden, das Risiko von Altersarmut steigt (Leitner 2022).

Die Alterung der Gesellschaft führt nicht nur in der Rentenversicherung zu Finanzierungsproblemen, sondern stellt auch das System der pflegerischen Versorgung vor neue Herausforderungen. Dies lässt sich an den seit Mitte der 1970er Jahre kontinuierlich steigenden Aufwendungen der kommunalen Sozialhilfehaushalte für Pflegeausgaben ablesen. Die Frage, wie die Versorgung einer zunehmenden Anzahl an Pflegebedürftigen bei gleichzeitig steigender Frauenerwerbstätigkeit zukünftig bewältigt werden kann, führte letztlich 1995 zur Einführung der Pflegeversicherung. Das Pflegegeld sollte die häusliche Pflege durch Angehörige stabilisieren, die Pflegesachleistung die Kosten für professionelle Versorgung subventionieren.^5^ Dadurch, dass die Leistungen pauschalisiert und nicht kostendeckend angelegt sind (Teilkaskoprinzip), müssen sich Pflegebedürftige und ihre Angehörigen an den Kosten der Pflege beteiligen. Im ambulanten Bereich geben 25 % der Pflegebedürftigen an, einen Eigenanteil in Höhe von durchschnittlich 250 € monatlich zu leisten. Insbesondere bei Pflegebedürftigen mit einer demenziellen Erkrankung und/oder einem Pflegegrad von 3 oder mehr kumulieren finanzielle, zeitliche und andere Belastungen in der häuslichen Pflege (Räker et al. 2020, S. 92 f.). Bei vollstationärer Pflege beträgt der Gesamteigenanteil im Bundesdurchschnitt 2100 € monatlich (Rothgang et al. 2020, S. 111). Etwa ein Drittel der Pflegebedürftigen und 44 % der pflegenden Angehörigen haben ein Einkommen unterhalb der Armutsgefährdungsschwelle (Rothgang und Müller 2018, S. 110) und sind für die Deckung von Pflegekosten in hohem Maße auf die Leistungen der Hilfe zur Pflege (SGB XII) angewiesen. Die Pflegeversicherung ist damit nur für einen Teil der Pflegebedürftigen und ihrer Angehörigen ein auskömmliches Leistungssystem.

Ebenfalls vor dem Hintergrund steigender Kosten – aufgrund der demographischen Entwicklung und wegen des medizinischen Fortschritts – wurde das Gesundheitssystem seit den 1990er Jahren zum Reformdauerbrenner. Beispielhaft soll hier als ein zentraler Versuch der Kostendämpfung das 2004 eingeführte Finanzierungsinstrument der Fallpauschalen (DRG – „diagnosis-related groups“) zur Deckelung der Kosten in der Krankenhausversorgung angeführt werden. Die Abrechnung nach Fallpauschalen anstatt nach konkreten Behandlungskosten setzt u. a. Anreize dafür, die Kosten des Einzelfalls niedriger zu halten als die Höhe der festgelegten Fallpauschale. Eher leichtere Fälle sind deshalb für Krankenhäuser attraktiver, ebenso eine möglichst kurze stationäre Verweildauer der Patient*innen. Dies stellt einen Anreiz dar, Leistungen implizit zu rationieren, indem schwere Fälle z. B. seltener aufgenommen oder zu früh in die ambulante Versorgung entlassen werden. Eine Vergütungsstruktur, die Verweildauern auf einen kritisch kurzen Zeitraum reduziert, setze Fehlanreize und gerade in der Kinder- und Jugendmedizin sowie bei der Versorgung multimorbider alter Patient*innen seien die Fallpauschalen erfahrungsgemäß zu gering bemessen, so der Deutsche Ethikrat (2016). Der Zielkonflikt zwischen einer hohen Qualität gesundheitlicher Versorgung und kostensparenden Steuerungsanreizen wird hier deutlich.

Ein nachlassendes Wirtschaftswachstum in Kombination mit höheren Ausgaben aufgrund der Alterung der Bevölkerung, steigender Arbeitslosigkeit und des medizinischen Fortschritts führte somit im Sozialversicherungssystem zur Absenkung des Standardrentenniveaus, zur Umverteilung der Kosten der Pflege durch die Einführung der Pflegeversicherung und zu Kostendämpfungsmaßnahmen im Gesundheitsbereich.^6^ Auch für die kommunale Sozialpolitik blieb die gesamtwirtschaftliche Entwicklung nicht ohne Folgen.

### Das Neue Steuerungsmodell der Kommunen

Die seit den 1990er Jahren forcierte Einführung des Neuen Steuerungsmodells (NSM) als Instrument der Effizienzsteigerung kommunaler Verwaltung trifft auch die Erbringungskontexte kommunaler Sozialpolitik, insbesondere die Einrichtungen und Dienste der Träger der (freien) Wohlfahrtspflege, die sich zunehmend nach betriebswirtschaftlichen Vorgaben ausrichten müssen und mehr und mehr in Konkurrenz zu privaten Leistungserbringern stehen (vgl. Klenk und Reiter 2012 für die Krankenhausinfrastruktur). Die Output-orientierte Steuerung über Zielvereinbarungen, der Wechsel vom Kostendeckungs- zum Budgetprinzip, ein ausführliches Controlling und Berichtswesen sowie die Einführung von Wettbewerbselementen in den *Dienstleistungsmarkt* markieren diese Entwicklung (Bogumil und Holtkamp 2013). Dadurch entsteht ein Spannungsfeld zwischen versorgungspolitischen und sparpolitischen Zielsetzungen, von dem – insbesondere unter dem Diktum von Kosteneinsparungen – negative Auswirkungen auf die Struktur und Qualität der sozialen Dienste sowie auf die betroffenen Bevölkerungsgruppen ausgehen (Grunow 2011). Im Rückblick kann konstatiert werden, dass das NSM nicht zu einem umfassenden Paradigmenwechsel im Sinne einer Modernisierung der Kommunalverwaltung geführt hat, sondern vielmehr die Thematik der Haushaltskonsolidierung das Verwaltungshandeln dominierte (Tabatt-Hirschfeld 2018). Erfahrungen mit dem NSM im Bereich der Jugendhilfe zeigten (Bogumil et al. 2007; Grohs 2010), dass die Umsetzung des NSM als zwiespältig bewertet werden muss: Zum einen erfolgte eine Verwaltungsmodernisierung durch Dezentralisierung, was aber oft eine größere Fragmentierung der Verwaltungsstrukturen hervorrief als vorher, da keine zentralen Steuerungsmechanismen etabliert wurden. Zum anderen haben sich Leistungsvereinbarungen und Kontraktmanagement durchaus auf breiter Linie in den Kommunen durchgesetzt, es kam dadurch aber nicht zu der beabsichtigten Pluralisierung der Anbieterstruktur. Schließlich wurden Kosteneinsparungen durch das Instrument der Budgetdeckelung zumeist ohne Rückkoppelung an sozialpolitische Zielsetzungen durchgesetzt, wodurch der Zielkonflikt einseitig zugunsten der Finanzierungsperspektive *gelöst* wurde. Soziale Dienstleistungen sind aber nur begrenzt marktfähig. Trotzdem wird von ihnen (betriebs)wirtschaftliches Handeln eingefordert, das oftmals im Widerspruch zur Fachlichkeit Sozialer Arbeit und ihren professionsethischen Grundsätzen steht (Hagn 2017).

Damit wird deutlich, dass es bei der Bewältigung der veränderten sozioökonomischen Rahmenbedingungen der Organisation des Sozialen immer auch um die ideologische Frage geht, welche Sicherungsziele aufrechterhalten werden sollen und von welchen man sich gegebenenfalls auch verabschiedet. In der Arbeitsmarktpolitik kann man dabei von einem Paradigmenwechsel sprechen.

### Die Hinwendung zum „adult worker model“

Infolge der ersten wirtschaftlichen Rezession der Nachkriegszeit 1967/68 wurde mit dem Arbeitsförderungsgesetz 1969 die Grundlage für eine aktive Arbeitsmarktpolitik geschaffen. Die Grundidee war, durch Beratung, Umschulungsangebote und Mobilitätsförderung die Strukturprobleme des Arbeitsmarktes zu beheben, d. h. Arbeitsangebot und Arbeitsnachfrage passend zu machen. Es handelte sich dabei stets um freiwillige Maßnahmen als Angebot für Arbeitslose zur Verbesserung ihrer Chancen auf dem Arbeitsmarkt. Arbeitslosigkeit wurde in Bezug auf das Gros der Erwerbstätigen nach wie vor als strukturelles Problem, mithin als soziales Risiko und nicht als individuell verschuldet verstanden.

Dies änderte sich mit dem zunehmenden Anstieg der Arbeitslosigkeit in den 1990er Jahren. Während es der Politik nicht gelang, Arbeitsplätze in ausreichendem Maße zu schaffen, wurden (insbesondere langzeit)arbeitslose Leistungsbeziehende immer deutlicher gesellschaftlich stigmatisiert. Die Legitimität bzw. die Rahmenbedingungen des Leistungsbezugs standen zur Diskussion, und damit rückte die Frage nach dem eigenen Verschulden an der individuellen Arbeitslosigkeit in den Vordergrund und die Strukturbedingungen des Arbeitsmarktes in den Hintergrund. Seinen gesetzgeberischen Ausdruck fand dieser Paradigmenwechsel 2005 mit der Einführung von Arbeitslosengeld II („Hartz IV“), was die zentrale Unterscheidung von erwerbsfähigen und nicht erwerbsfähigen Bedürftigen im Bereich der Grundsicherungsleistungen mit sich brachte. Damit wurde eine neue Differenzierungslinie der Leistungsgewährung eingeführt, die einem neuen Leitbild folgt: dem „adult worker model“ (Lewis 2001).

Dieses produktivistische Paradigma geht davon aus, dass alle, die erwerbsfähig sind, auch erwerbstätig sein sollen. Nur die nicht erwerbsfähigen Bedürftigen können ungefragt und über einen langen Zeitraum hinweg Grundsicherungsleistungen beziehen. Die Erwerbsfähigen hingegen müssen sich auf das Prinzip „Keine Leistung ohne Gegenleistung“ bzw. „Fördern und Fordern“ einstellen. Grundsätzlich werden Langzeitarbeitslose verpflichtet, jede Arbeit anzunehmen, die nicht sittenwidrig ist; die Frage der Zumutbarkeit wird kaum gestellt, es gibt keinen Berufsschutz mehr. Bei Verstoß gegen die Anforderungen der Arbeitsagentur drohen Sanktionen. Aus einer aktiven ist eine aktivierende und sanktionierende Arbeitsmarktpolitik geworden.

Damit verbunden ist auch eine aktivierungspolitische Instrumentalisierung der Sozialen Arbeit: Sozialpädagogische Angebote werden zunehmend mit einem Fokus auf das Ziel der Beschäftigungsförderung ausgerichtet. Der sozialpädagogische Anspruch der ganzheitlichen Förderung von Menschen in schwierigen Lebenslagen gerät mehr und mehr in den Hintergrund bzw. wird marginalisiert. Lutz (2018) spricht in diesem Zusammenhang von einer Rückkehr der Konzepte *Schuld und Moral* sowie von Instrumenten der Kontrolle, Repression und Ausschließung. Die neue Pathologie heißt In-Aktivität.

### Die Diversifizierung des männlichen Ernährermodells in der Familienpolitik

Ein Leitbildwandel anderer Art ist in der Familienpolitik festzustellen. Mit dem Anstieg der (westdeutschen) Frauenerwerbstätigkeit seit den 1970er Jahren stellte sich zunehmend die Frage nach der Vereinbarkeit von Kindererziehung und Beruf, die vom Wohlfahrtsstaat 1986 mit der Einführung von Erziehungsurlaub und Erziehungsgeld beantwortet wurde. Die neuen Leistungen waren auf eine „modifizierte männliche Ernährerehe“ (vgl. Pfau-Effinger 1996) zugeschnitten, d. h. das Geschlechterleitbild hatte sich insofern gewandelt, als dass Frauen nunmehr auch als Erwerbstätige konstruiert wurden. Allerdings galt die weibliche Erwerbstätigkeit über den Lebenslauf hinweg als variabel: Vor der Ehe bzw. der Geburt des ersten Kindes sollten Frauen in Vollzeit erwerbstätig sein, dann folgte eine mehr oder weniger lange Auszeit zum Zweck der Kindererziehung, und wenn das jüngste Kind im Kindergartenalter war, stand ein Wiedereinstieg in den Arbeitsmarkt auf Teilzeitbasis auf dem Programm, der aber auch zugunsten von Angehörigenpflege entfallen konnte. Die Erwerbstätigkeit der Frau war als *Zuverdienst* für die Familie markiert, die Vorrangstellung des männlichen Ernährers und die Zuständigkeit von Frauen für die Familienarbeit blieben zunächst unangetastet.

Erst mit dem Ausbau der Kinderbetreuung im U3-Bereich (seit 2005) und der Einführung des Elterngelds 2007 werden Mütter verstärkt als Erwerbstätige und Väter als Erziehende in den Blick genommen.^7^ Ziel war mittelfristig die Erhöhung der Müttererwerbstätigkeit und langfristig die Erhöhung der Geburtenrate, um zukünftig drohendem Arbeitskräftemangel vorzubeugen. Der rasche Wiedereinstieg nach dem ersten Geburtstag des Kindes, mithin die Orientierung am „adult worker model“, sowie die Vorstellung einer partnerschaftlich geteilten Elternschaft wird strukturell als gesellschaftliche Erwartungshaltung an Eltern verankert. Kinderbetreuung wird durch den neuen Rechtsanspruch auf einen Betreuungsplatz ab dem ersten Lebensjahr aus der Familie ausgelagert, um die Eltern potenziell für den Arbeitsmarkt freizustellen. Für Männer/Väter wie für Frauen/Mütter gilt nunmehr das Primat der Erwerbsarbeit. Erziehungsarbeit wird für beide Geschlechter nur in engen zeitlichen Schranken honoriert.

Die traditionelle Ehezentrierung der deutschen Sozialpolitik blieb gleichzeitig in Form der beitragsfreien Mitversicherung, der Hinterbliebenenrenten und des Ehegattensplittings erhalten und bietet somit widersprüchliche Anreize für die Gestaltung des familialen Geschlechterverhältnisses. Positiv gewendet, könnte man auch von einer größeren Wahlfreiheit für Eltern sprechen, wenngleich diese immer durch die jeweiligen Rahmenbedingungen, wie z. B. Höhe des Familieneinkommens, Verfügbarkeit von Betreuungsplätzen oder individuelle Arbeitsmarktchancen, eingeschränkt bleibt.

### Neujustierung und Neuerfindung des Sozialen

Die beschriebenen Veränderungsprozesse in Bezug auf die Finanzierung und die normativen Sicherungsziele der Sozialpolitik weisen darauf hin, dass der Wandel der sozialen Sicherungssysteme nicht einheitlich in Richtung eines *liberalen* Wohlfahrtsregimes erfolgt. Vielmehr ist eine Ausdifferenzierung der Institutionen und Instrumente zu beobachten, die unterschiedlichen – teils miteinander im Widerspruch stehenden – Leitbildern folgt. Kessl (2013) spricht in diesem Zusammenhang zeitdiagnostisch von der „Neujustierung des wohlfahrtsstaatlichen Arrangements“, Lessenich (2008) von der „Neuerfindung des Sozialen“. Als zentrale Paradigmen der jüngeren Reformpolitiken können dabei folgende identifiziert werden:

- Das Aktivierungsparadigma zielt auf die (Re‑)Integration in den Arbeitsmarkt ab und manifestiert sich entweder direkt im Rahmen sozialer Dienstleistungen oder indirekt über den Wegfall, die Kürzung oder den zeitlichen Aufschub monetärer (*passiver*) Sozialleistungen. Hierunter fällt vor allem die Aktivierung von älteren Menschen (für den Arbeitsmarkt oder das Ehrenamt), von erwerbsfähigen Grundsicherungsbeziehenden, von erwerbsorientierten Eltern sowie von Jugendlichen in der Vorerwerbsphase (durch Praktika und Bildungszertifikate). Aktivierung und das „adult worker model“ gehen Hand in Hand.
- Das Eigenverantwortungsparadigma überträgt allen Bürger*innen Verantwortung zur Vorsorge und Selbstorganisation in Bezug auf die eigene Gesundheit, Erwerbsfähigkeit, Altersvorsorge u. v. a. Dazu gehört etwa die eigenverantwortliche Vorsorge für das Alter durch den Abschluss einer Riester- oder Rürup-Rente, die Zuzahlungen von Kranken und Pflegebedürftigen zu den Behandlungs- bzw. Pflegekosten, die Selbstverpflichtung im Rahmen des Grundsatzes „Fordern und Fördern“, die Bereitschaft zum lebenslangen Lernen und den Erwerb von Bildungszertifikaten unter dem Aspekt der Verwertbarkeit für den Arbeitsmarkt oder auch die Einführung von so genannten Bonus-Malus-Systemen im Bereich der Hilfen zur Erziehung.
- Das Autonomie- bzw. Selbstbestimmungsparadigma räumt den Adressat*innen (scheinbare) Wahl- und Mitbestimmungsmöglichkeiten ein. Dies wird beispielsweise ermöglicht durch die eigenständige Wahl des privaten Altersvorsorgeprodukts oder der Krankenkasse, die Ermöglichung von Wahlfreiheit zwischen häuslicher und öffentlicher Kinderbetreuung für Eltern oder die Wahloption zwischen ambulanter, teilstationärer oder stationärer Pflege von Älteren. Auch die Mitsprachemöglichkeiten von Patent/innen am Behandlungsprozess im Sinne eines „shared decision-making“ oder die Ausstattung von Pflegebedürftigen oder von behinderten Menschen mit einem individuellen Pflege- bzw. einem persönlichen Budget, über das sie selbst bestimmen können, sind Maßnahmen, die unter diesem Stichwort diskutiert werden.

Das Soziale ist im deutschen Wohlfahrtsstaat traditionell entlang der Prinzipien Erwerbsorientierung, Ehezentrierung und ergänzender bedarfsorientierter Existenzsicherung organisiert. Auf die sozioökonomischen Herausforderungen einer sich verschlechternden wirtschaftlichen Lage bei gleichzeitigem Anstieg der Sozialausgaben reagierte die Sozialpolitik mit einer Modifikation von Sicherungszielen und veränderten Leitbildern in Bezug auf Arbeitslosigkeit, Erwerbstätigkeit und Kindererziehung. Diese Bewältigungsweisen können mit den übergreifenden Paradigmen der Aktivierung, der Eigenverantwortung und der Selbstbestimmung beschrieben werden. Damit stellt sich grundsätzlich die Frage, inwiefern der deutsche Wohlfahrtsstaat in seiner Gesamtheit noch in der Lage ist, soziale Problemlagen aufzufangen. Aus einer ganzheitlichen Perspektive wird dafür plädiert, die Fragmentierung des sozialen Sicherungssystems zu überwinden und von konkreten Problemkonstellationen aus zu denken.

## Konturen einer integrierten Perspektive auf das Soziale

Betrachtet man nun das Soziale aus einer ganzheitlichen Perspektive, stehen zunächst nicht die Organisationsstrukturen, sondern die Problemkonstellationen im Vordergrund. Zu fragen wäre danach, welche Bedarfslagen durch welche Leistungen abgedeckt werden können, wo es zu Schnittstellenproblematiken kommt bzw. Leistungen aus einer Hand erforderlich wären und wo ggf. Sicherungslücken bestehen. Ein Beispiel soll dies veranschaulichen: Erwerbsfähige Langzeitarbeitslose und ihre Familien können eine Reihe von unterschiedlichen Bedarfen aufweisen. Neben dem offensichtlichen Bedarf der materiellen Existenzsicherung kann die Notwendigkeit der Qualifizierung oder Umschulung bestehen, oder es können Reha-Maßnahmen angezeigt sein; Kinder brauchen eventuell Unterstützung durch Nachhilfe, psychosoziale Dienste oder Integrationsmaßnahmen; Kinderbetreuung oder ambulante pflegerische Dienste können die Erwerbstätigkeit beider Elternteile unterstützen. Für die Organisation dieser Leistungen sind jeweils unterschiedliche Institutionen und Verwaltungsebenen zuständig: die Arbeitslosenversicherung, die Grundsicherung, die Unfall- oder Krankenkasse, das Jobcenter, das Sozialamt, das Integrationsamt, das Jugendamt, die Pflegeberatung; und es erfordert eine gewisse Selbstorganisationsfähigkeit, die entsprechenden Leistungen zu identifizieren und einzufordern. Je komplexer die Bedarfslage eines Individuums ist, desto größer ist die Wahrscheinlichkeit, dass (gleichzeitig oder nacheinander) sozialpolitische Leistungen aus unterschiedlichen Rechtskreisen benötigt werden und dass eine Vielzahl an Akteur*innen an der Bearbeitung der Risikosituation beteiligt ist. Auf Ebene der Leistungserbringer erfolgt zumeist keine Kooperation in Bezug auf den Einzelfall, sodass es sowohl zu Über- als auch zu Unterversorgungslagen sowie zu Fehlversorgung kommen kann. Bogumil und Kuhlmann (2022) beschreiben diese Phänomene als Probleme der horizontalen und vertikalen Verwaltungsverflechtung im Mehrebenensystem, die zu Schnittstellenproblemen als Ausdruck von Überflechtung, Unterflechtung und Koordinationsmängeln führen. Zudem werden auf der Makroebene Fragen der Finanzierung und der Sicherungsziele übergreifend verhandelt, wobei es durchaus zu Konkurrenzen zwischen den Leistungsbereichen und widersprüchlichen Sicherungszielen kommen kann. Diese Fragmentierung der Sozialpolitik stellt dann wiederum die Adressat*innen vor Passungsprobleme, weil sie mit sozialen Problemen konfrontiert sind, die sich nicht an vorgegebene Verwaltungsstrukturen halten und ein umfassendes Schnittstellenmanagement erfordern. Eine multiperspektivische Betrachtungsweise der Organisation des Sozialen muss eine ressortübergreifende Querschnittsperspektive einnehmen und die Mikro‑, Meso- und Makroebene der Sozialpolitik verknüpfen.

Ausgehend von den Adressat*innen staatlicher Sozialpolitik und ihren Problemlagen stellen sich auf einer Mikroebene Fragen der Zugänglichkeit, Niedrigschwelligkeit und Diskriminierungsfreiheit von Leistungen. Welche Barrieren der Inanspruchnahme bestehen? Sind die Leistungen bedarfsdeckend? Wie gestaltet sich der *Gebrauchswert* sozialer Leistungen aus Nutzer*innenperspektive? Inwiefern kann durch partizipative Verfahren einer Wohlfahrtsproduktion *von unten* eine Verbesserung der *Passung* von Leistungen erreicht werden? Wie kann die Selbstvertretung von Adressat*innen gefördert werden? Eine an individuellen Lebenslagen und Risikosituationen orientierte Sozialpolitik erfordert die aktive Einbeziehung der Adressat*innen bzw. Nutzer*innen sowie von Implementationserfahrungen der Dienstleistungserbringer in die Politik(re)formulierung. Hier kommt der advokatorischen Interessenvertretung durch Soziale Arbeit eine wichtige Rolle zu, erstrebenswert(er) erscheint die durch Empowermentprozesse zu unterstützende Selbstvertretung von Betroffenen. Während im Bereich der Behindertenpolitik bereits eine Vielzahl an Betroffenenorganisationen versucht, sich Gehör zu verschaffen – was etwa im Zuge des Politikprozesses zum Bundesteilhabegesetz deutlich wurde (vgl. Schultz 2022) – scheint es für Armutsbetroffene schwieriger zu sein, sich zu organisieren. Positiv zu nennen sind hier das Projekt „Beteiligung von Menschen mit Armutserfahrung“ der Diakonie, die Koordinierungsstelle gewerkschaftlicher Arbeitslosengruppen, das Treffen der Menschen mit Armutserfahrung im Rahmen der Nationalen Armutskonferenz oder das Erwerbslosenparlament in Mecklenburg-Vorpommern. Derartige Initiativen sollten noch stärker unterstützt werden, um das Soziale aus Betroffenenperspektive mitzugestalten. Es gibt dahingehend auch erste ermutigende Signale aus der Sozialverwaltung: Das Jobcenter Köln hat jüngst einen Kund*innenbeirat ins Leben gerufen, der Vorschläge zur Verbesserung der Angebote, der Kommunikation und der Services des Jobcenters machen soll.

Auf der Mesoebene der konkreten Organisation von Leistungen bei Sozialversicherungsträgern, Einrichtungen der Wohlfahrtsverbände und Kommunen stellen sich Fragen zum organisationalen Setting. Die Erbringungsbedingungen für die Beschäftigten im Dienstleistungsbereich stellen sich nach wie vor kritisch dar: Eine hohe Arbeitsbelastung steht einer mangelnden (finanziellen) Anerkennung gegenüber, fachliche Erfordernisse geraten in Konflikt mit betriebswirtschaftlichen Vorgaben (vgl. Henn et al. 2017). Unter diesen Bedingungen ist ein am Einzelfall orientiertes Schnittstellenmanagement kaum zu stemmen. Das heißt im Umkehrschluss, dass für die Bewältigung von Schnittstellenproblemen, respektive in Form von Case-Management, zusätzliche Ressourcen eingeplant werden müssen. Eine konsequente Orientierung an sozialen Problemlagen erfordert deshalb zunächst einen höheren finanziellen Aufwand, um passgenauere Lösungen zu erzielen. Dies mag Versuchen der Kostendämpfung im Sozialbereich entgegenstehen, ist aber für eine ganzheitliche Organisation des Sozialen notwendig. Ein Outsourcing sozialer Dienstleistungen als unbezahlte Tätigkeiten in Vereinen, Nachbarschaften, Projekten oder sozialen Netzwerken kann dafür jedenfalls keine Lösung darstellen. So wertvoll zivilgesellschaftliches Engagement und Freiwilligenarbeit sind: Sie basieren auf persönlichen Beziehungen und Abhängigkeiten, nicht auf sozialen Rechtsansprüchen (vgl. van Dyk und Haubner 2021). Ebenso abzulehnen ist in diesem Sinne die staatliche Förderung von gewinnorientierten Organisationen der sozialen Dienstleistungserbringung. Demgegenüber stünde das Erfordernis der Gemeinnützigkeit von sozialen Einrichtungen und Diensten sowie der Reinvestition von Gewinnen in die Qualität der Dienstleistungserbringung.

Auf der Makroebene stellen sich Fragen nach den gesamtgesellschaftlichen Rahmenbedingungen staatlicher Sozialpolitik. Inwiefern ist das Prinzip der Lohnarbeitszentrierung noch zeitgemäß? Kann die kompensatorische und subsidiäre Logik der Sozialpolitik angesichts veränderter Arbeits- und Lebensweisen noch aufrechterhalten werden? Hier ist der Vorschlag, Sozialpolitik als soziale Infrastruktur zu denken nach wie vor überlegenswert (vgl. AG links-netz 2003). In diesem Sinne müsste das Angebot an öffentlichen Gütern und Dienstleistungen grundsätzlich erweitert werden. Grundbedürfnisse in den Bereichen Gesundheit, Bildung, Verkehr und Wohnen müssten rechtsverbindlich und kostenlos oder zumindest kostengünstig abgedeckt werden. Soziale Dienstleistungen müssten zudem neu und von ihren Gebrauchswerten her bestimmt werden. Es stellen sich damit normative Fragen, welche Sicherungsziele langfristig verfolgt werden und wie das Soziale zwischen den polarisierenden Perspektiven einer staatlichen Steuerung der Lebensverhältnisse und einer Gestaltung der Lebensverhältnisse durch die Subjekte selbst (im Sinne einer „Politik des Sozialen“, vgl. Widersprüche 97/2005) verfasst sein soll.

Ein sozialpolitisches Forschungsprogramm, das das Soziale aus einer ganzheitlichen Perspektive in den Blick nimmt, muss von individuellen Problemlagen aus denken und erfordert ein integrierendes Verständnis der Mikro‑, Meso- und Makroebene des Sozialen, um die Fragmentierung der Sozialpolitik zu überwinden.

## Footnotes

1 Für die historischen Grundlegungen wird auszugsweise auf Leitner (2017) zurückgegriffen.

2 Allerdings war strittig, ob die Gewerkschaften oder der Staat die neu entstehende Arbeitslosenversicherung organisieren sollte.

3 Beim Ehegattensplitting werden die Einkommen beider Ehepartner*innen addiert und durch zwei dividiert. Die dadurch errechneten (gleich hohen) individuellen Einkommen werden je für sich besteuert. In einem progressiven Steuersystem verringert sich somit die Steuerlast, wenn die Ehepartner*innen sehr ungleiche Erwerbseinkommen haben. Am meisten profitieren Alleinverdiener-Ehepaare.

4 Die beitragsfreie Mitversicherung von nicht erwerbstätigen Ehepartner*innen ist seit 1930 (sg. Familienhilfe) geschlechtsneutral geregelt, die Hinterbliebenenrente seit 1986. Somit erlaubt der deutsche Wohlfahrtsstaat im Prinzip einen Rollentausch innerhalb der Ehegemeinschaft. De facto werden diese Sicherungselemente aber bis heute in weitaus höherem Ausmaß für Frauen relevant, während Männer überwiegend über die eigene Erwerbstätigkeit abgesichert sind.

5 Es ist an der Stelle bemerkenswert, dass die Pflegeversicherung nicht die Pflegenden im Sinne einer Lohnersatzleistung für den (teilweisen) Ausfall des Erwerbseinkommens absichert, sondern sich rein auf die pflegerische Versorgungsleistung fokussiert. Damit unterscheidet sie sich fundamental von der Krankenversicherung, die sowohl einen Lohnersatz als auch medizinische Versorgung beinhaltet.

6 Es könnten hier noch weitere Einsparungsmaßnahmen in den unterschiedlichen Sozialversicherungszweigen wie die Erhöhung von Beitragssätzen und Kürzungen im Leistungsbezug angeführt werden, deren detaillierte Darstellung hier zu weit führen würde (siehe dazu Bäcker 2021).

7 Das Elterngeld ist zwar als Lohnersatzleistung konzipiert, aber nicht als Elternschaftsversicherung. Die Finanzierung erfolgt über Steuergelder und nicht über Versicherungsbeiträge, wie dies z. B. in Schweden der Fall ist.
